# Crystal structure of 4,4′-bis­(4-bromo­phen­yl)-1,1′,3,3′-tetra­thia­fulvalene

**DOI:** 10.1107/S2056989019009952

**Published:** 2019-07-16

**Authors:** Sergei Rigin, Marina Fonari

**Affiliations:** a Department of Chemistry, New Mexico Highlands University, Las Vegas, New Mexico, 87701, USA

**Keywords:** crystal structure, tetra­thia­fulvalene, derivative, weak inter­actions, Hirshfeld surface analysis, DFT calculations

## Abstract

The mol­ecule of the title compound, C_18_H_10_Br_2_S_4_, has a C-shape, with *C*_s_ mol­ecular symmetry. The dihedral angle between the planes of the di­thiol and phenyl rings is 8.35 (9)°. In the crystal, mol­ecules form helical chains along [001], the shortest inter­actions being π⋯S contacts within the helices. The inter­molecular inter­actions were investigated by Hirshfeld surface analysis. Density functional theory (DFT) was used to calculate HOMO–LUMO energy levels of the title compound and its *trans* isomer.

## Abstract 2

The mol­ecule of the title compound has a C-shape, with *C*_s_ mol­ecular symmetry. The dihedral angle between the planes of the di­thiol and phenyl rings is 8.35 (9)°. In the crystal, mol­ecules form helical chains along [001], the shortest inter­actions being π⋯S contacts within the helices.

## Chemical context   

So far significant progress has been achieved in improving the performance of organic field-effect transistors (OFETs) using such materials as oligoacenes, oligo­thio­phenes and polythio­phenes (Mas-Torrent & Rovira, 2011 ▸; Pfattner, *et al.*, 2016 ▸). Numerous derivatives of the sulfur heterocycle 2,2′-bis­(1,3-di­thio­lyl­idene), known as tetra­thia­fulvalene (TTF), have been noted as components of OFETs (Fourmigué & Batail, 2004 ▸; Bendikov *et al.*, 2004 ▸). High charge mobilities have been reported for thio­phene-fused TTF and dibenzo-TTF in single-crystal OFETs obtained from solutions, as well as in tetra(octa­decyl­thio)-TTF films (Mas-Torrent *et al.*, 2004*a* ▸,*b* ▸). A comparatively high mobility was reported for biphenyl-substituted TTF (Noda *et al.*, 2005 ▸, 2007 ▸). Correlations between mobilities and herring-bone crystal structures have been investigated (Pfattner, *et al.*, 2016 ▸; Mas-Torrent & Rovira, 2011 ▸), including for phenyl-substituted oligo­thio­phenes (Noda *et al.*, 2007 ▸). Among the numerous reported halogenated tetra­thia­fulvalenes (Fourmigué & Batail, 2004 ▸), only a few have been crystallographically characterized. The synthesis and characterization of two halogen TTF derivatives, 4,4′-bis­(4-chloro­phen­yl)tetra­thia­fulvalene and 4,4′-bis­(4-bromo­phen­yl)tetra­thia­fulvalene have been reported, but only the crystal structure of the chloro-substituted compound has been documented (Madhu & Das, 2008 ▸), which shows short Cl⋯Cl contacts. Herein, we report the crystal structure, the Hirshfeld surface analysis and the mol­ecular orbital analysis of the title compound, 4,4′-bis­(4-bromo­phen­yl)-1,1′,3,3′-tetra­thia­fulvalene (BBP-TTF).
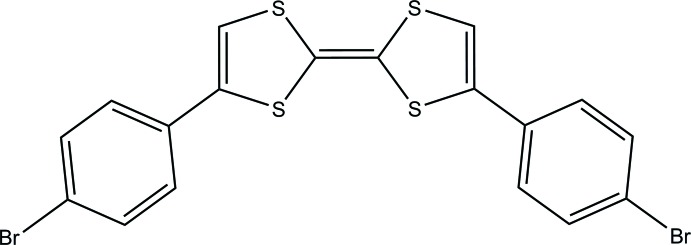


## Structural commentary   

**Figure 1:**
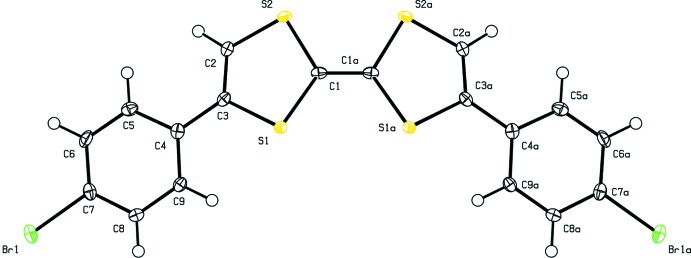
A view of the mol­ecular structure of the title compound with the atom labelling. Displacement ellipsoids are drawn at the 50% probability level. Suffix a corresponds to the symmetry operation *x*, −*y* + 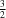, *z*.

The mol­ecular structure of the title compound is illustrated in Fig. 1 ▸. The mol­ecule has a C-shape with *C*_s_ mol­ecular symmetry and resides on the mirror plane passing through the central C1=C1(*x*, −y + 3/2, *z*) bond [1.343 (7) Å]. The C—S distances in the TTF moiety are in the range 1.729 (4)–1.778 (4) Å and correspond to reported values (CSD version 5.40, last update November 2018; Groom *et al.*, 2016 ▸). The dihedral angle between the di­thiol and phenyl rings is 8.35 (9)°.

## Supra­molecular features   

**Figure 2:**
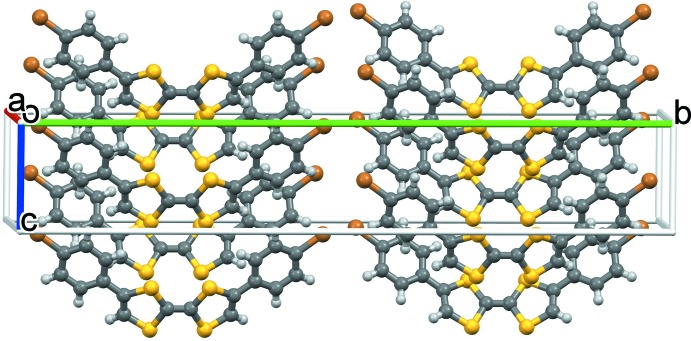
The crystal packing of the title compound.

In the crystal (Fig. 2 ▸), no significant inter­molecular inter­actions were found. Mol­ecules related by the twofold screw axis form helices along the *c-*axis direction. The dihedral angle between the mean planes of the adjacent mol­ecules in the helix is 36.59 (3)° and the helical pitch is 6.1991 (5) Å. The shortest inter­actions within the chain, as indicated by *Mercury* (Macrae *et al.*, 2006 ▸), are the S⋯π contacts C3⋯S2(1 − *x*, *y*, *z* − 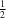) = 3.458 (4) and C2⋯S2(1 − *x*, *y*, *z* − 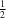) = 3.465 (4) Å, followed by the C2—H2⋯C4(1 − *x*, *y*, 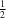 + *z*) [2.72, 3.467 (5) Å] short contacts that are in agreement with the Hirshfeld (1977 ▸) surface analysis.

## Hirshfeld surface analysis   

**Figure 3:**
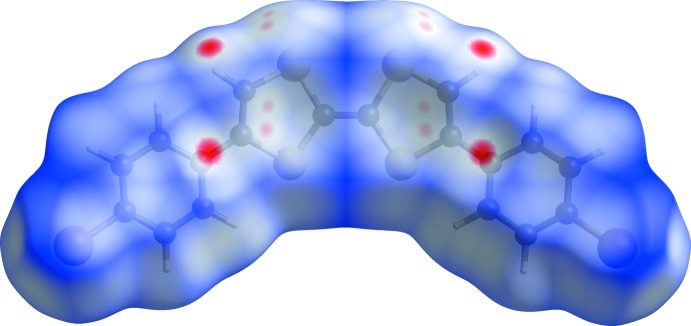
Hirshfeld surface mapped over *d*_norm_ for the title compound in the range −0.1138 to 1.1257 a.u.

*CrystalExplorer17.5* (Wolff *et al.*, 2012 ▸, Mackenzie *et al.*, 2017 ▸) was used to generate the mol­ecular Hirshfeld surface. The total *d*_norm_ surface of the title compound is shown in Fig. 3 ▸ where the red spots correspond to the most significant inter­actions in the crystal. In the studied mol­ecule, they include only weak C—H⋯π inter­actions at distances that are slightly higher than the sum of van der Waals radii.

## Frontier mol­ecular orbital calculations   

**Figure 4:**
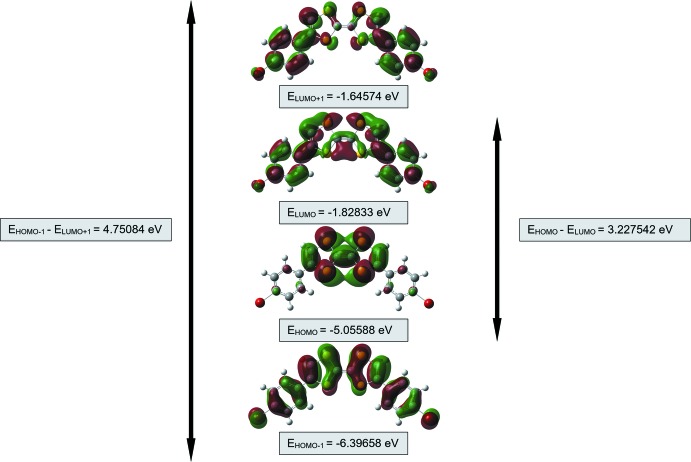
Mol­ecular orbital energy levels of the title compound (*cis* isomer).

The highest occupied mol­ecular orbital (HOMO) acts as an electron donor and the lowest unoccupied mol­ecular orbital (LUMO) acts as an electron acceptor. A small HOMO–LUMO energy gap indicates a highly polarizable mol­ecule and high chemical reactivity. Mol­ecular orbital energy levels for the title compound were calculated with *Gaussian 16W* software (Frisch *et al.*, 2016 ▸) using density functional theory (DFT) at the B3LYP/6-311+G(d,p) level of theory. The frontier orbitals of the title compound and its *trans*-isomer are shown in Figs. 4 ▸ and 5 ▸, respectively. The energy gap determines chemical hardness, chemical potential, electronegativity and the electrophilicity index. The orbital energy values for the title compound, its *trans*-isomer and unsubstituted TTF are summarized in Table 1 ▸. The conformation energy difference between the *cis*- and *trans* isomers is 1.6331 kJ mol^−1^. For both isomers the energy gap is large; hence both mol­ecules are considered to be hard materials and would be difficult to polarize. As seen from Table 1 ▸, the bromo­phenyl substituents reduce the HOMO–LUMO energy gap and therefore the unsubstituted TTF mol­ecule would be even more difficult to polarize.

## Database survey   

A search of the Cambridge Structural Database (CSD version 5.40, last update November 2018, Groom *et al.*, 2016 ▸) for substituted TTF-phenyl derivatives related to the title compound yielded six structures. They include: bis­(4,4′-di­phenyl­tetra­thia­fulvalenium)bis­(penta­fluoro­phen­yl)gold(I) (CAKTAJ; Cerrada *et al.*, 1998 ▸), 4,5′-di­phenyl­tetra­thia­fulvalene (DPTFUL; Escande & Lapasset, 1979 ▸, and DPTFUL01; Noda *et al.*, 2007 ▸), 4,4′-bis­(4-chloro­phen­yl)-1,1′,3,3′-tetra­thia­fulvalene (GOBVUP; Madhu & Das, 2008 ▸), 4,5′-bis­(*p*-tol­yl)tetra­thia­fulvalene (MOPJOR; Noda *et al.*, 2007 ▸), 4,5′-bis­(4-ethyl­phen­yl)tetra­thia­fulvalene (MOPJUX; Noda *et al.*, 2007 ▸), and 4,5′-bis­(4-(tri­fluoro­meth­yl)phen­yl)tetra­thia­fulvalene (MOPKEI; Noda *et al.*, 2007 ▸). Contrary to the title compound, they all exhibit inversion or pseudo-inversion symmetry with a *trans*-arrangement of the phenyl substituents about the central C=C bond. The C=C bond lengths vary from 1.339 Å (MOPJUX) to 1.353 Å (DPTFUL); the value observed for the title compound falls within this limit. All of the above mol­ecules are almost planar, with tilt angles between the di­thiol and phenyl rings varying from 5.39 to 10.18° for the two independent mol­ecules in DPTFUL01 to 28.28° in GOBVUP and 30.29° in MOPKEI; the greatest twisting was observed for halogen-substituted derivatives.

## Crystallization   

The single crystals of the title compound were obtained in attempt to co-crystallize it with tetra­cyano­quinodi­methane (TCNQ) in a 1:1 molar ratio. A saturated solution of 4,4′-bis­(4-bromo­phen­yl)-1,1′,3,3′-tetra­thia­fulvalene (2 mg, Aldrich) in chloro­form was mixed with a saturated solution of TCNQ (1 mg, Aldrich) in aceto­nitrile and left at room temperature. Red prismatic crystals suitable for the X-ray diffraction analysis were obtained after a week of slow evaporation.

## Refinement   

Crystal data, data collection and structure refinement details are summarized in Table 2 ▸. The hydrogen atoms were positioned geometrically and refined using a riding model: C—H = 0.93 Å with *U*_iso_(H) = 1.2*U*_eq_(C).

## Supplementary Material

Crystal structure: contains datablock(s) I. DOI: 10.1107/S2056989019009952/eb2019sup1.cif

Structure factors: contains datablock(s) I. DOI: 10.1107/S2056989019009952/eb2019Isup2.hkl

Supporting information file. DOI: 10.1107/S2056989019009952/eb2019Isup3.cml

CCDC reference: 1940080

Additional supporting information:  crystallographic information; 3D view; checkCIF report

## Figures and Tables

**Figure 5:**
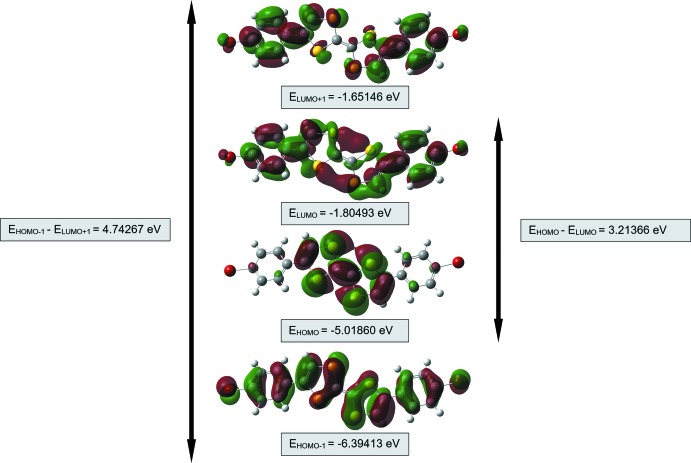
Mol­ecular orbital energy levels of the *trans* isomer of the title compound.

**Table 1:** Calculated frontier mol­ecular orbital energies (eV) for the title compound, its *trans* isomer and unsubstituted TTF and the conformational energy differences (kJ mol^−1^) between the *cis* and *trans* isomers

	*cis* isomer	*trans* isomer	TTF
*E*(HOMO)	−5.0559	−5.0186	−4.8488
*E*(LUMO)	−1.8283	−1.8049	−1.1252
*E*(HOMO-1)	−6.3966	−6.3941	−6.6303
*E*(LUMO+1)	−1.6457	−1.6515	−0.7140
Δ*E*(HOMO–LUMO)	3.2275	3.2137	3.7236
Δ*E*(HOMO-1–LUMO+1)	4.7508	4.7427	5.9163
			
Chemical hardness (η)	1.6138	1.6068	1.8618
Chemical potential (μ)	3.4421	3.4118	2.9870
Electronegativity (χ)	−3.4421	−3.4118	−2.9870
Electrophilicity index (ω)	3.6709	3.6221	2.3961
			
Δ*E*(*cis–trans*)	1.6331		

**Table 2:** Experimental details

Crystal data	
Chemical formula	C_18_H_10_Br_2_S_4_
*M*_r_	514.32
Crystal system, space group	Orthorhombic, *A* *b* *m*2
Temperature (K)	90
*a*, *b*, *c* (Å)	7.5981 (6), 37.411 (3), 6.1991 (5)
*V* (Å^3^)	1762.1 (2)
*Z*	4
Radiation type	Mo *K*α
μ (mm^−1^)	5.07
Crystal size (mm)	0.17 × 0.11 × 0.05

Data collection	
Diffractometer	Bruker APEXII CCD
Absorption correction	Multi-scan (*SADABS*; Bruker, 2016 ▸)
*T*_min_, *T*_max_	0.625, 0.747
No. of measured, independent and observed [*I* > 2σ(*I*)] reflections	34235, 1580, 1530
*R*_int_	0.066
(sin θ/λ)_max_ (Å^−1^)	0.594

Refinement	
*R*[*F* ^2^ > 2σ(*F* ^2^)], *wR*(*F* ^2^), *S*	0.017, 0.041, 1.09
No. of reflections	1580
No. of parameters	109
No. of restraints	1
H-atom treatment	H-atom parameters constrained
Δρ_max_, Δρ_min_ (e Å^−3^)	0.29, −0.29
Absolute structure	Flack *x* determined using 663 quotients [(*I* ^+^)−(*I* ^−^)]/[(*I* ^+^)+(*I* ^−^)] (Parsons et al., 2013 ▸)
Absolute structure parameter	0.014 (5)

Computer programs: *APEX2* and *SAINT* (Bruker, 2016 ▸), *SHELXT* (Sheldrick, 2015*a* ▸), *SHELXL2018* (Sheldrick, 2015*b* ▸) and *OLEX2* (Dolomanov *et al.*, 2009 ▸).

## supplementary crystallographic information

### Crystal data

**Table:**

C_18_H_10_Br_2_S_4_	*D*_x_ = 1.939 Mg m^−^^3^
*M**_r_* = 514.32	Mo *K*α radiation, λ = 0.71073 Å
Orthorhombic, *A**b**m*2	Cell parameters from 9390 reflections
*a* = 7.5981 (6) Å	θ = 2.2–28.4°
*b* = 37.411 (3) Å	µ = 5.07 mm^−^^1^
*c* = 6.1991 (5) Å	*T* = 90 K
*V* = 1762.1 (2) Å^3^	Prism, red
*Z* = 4	0.17 × 0.11 × 0.05 mm
*F*(000) = 1008	

### Data collection

**Table:**

Bruker APEXII CCD diffractometer	1530 reflections with *I* > 2σ(*I*)
φ and ω scans	*R*_int_ = 0.066
Absorption correction: multi-scan (SADABS; Bruker, 2016)	θ_max_ = 25.0°, θ_min_ = 1.1°
*T*_min_ = 0.625, *T*_max_ = 0.747	*h* = −9→9
34235 measured reflections	*k* = −44→44
1580 independent reflections	*l* = −7→7

### Refinement

**Table:**

Refinement on *F*^2^	Hydrogen site location: inferred from neighbouring sites
Least-squares matrix: full	H-atom parameters constrained
*R*[*F*^2^ > 2σ(*F*^2^)] = 0.017	*w* = 1/[σ^2^(*F*_o_^2^) + (0.0126*P*)^2^ + 2.1911*P*] where *P* = (*F*_o_^2^ + 2*F*_c_^2^)/3
*wR*(*F*^2^) = 0.041	(Δ/σ)_max_ = 0.003
*S* = 1.09	Δρ_max_ = 0.29 e Å^−^^3^
1580 reflections	Δρ_min_ = −0.29 e Å^−^^3^
109 parameters	Absolute structure: Flack *x* determined using 663 quotients [(*I*^+^)-(*I*^-^)]/[(*I*^+^)+(*I*^-^)] (Parsons et al., 2013)
1 restraint	Absolute structure parameter: 0.014 (5)
Primary atom site location: dual	

### Special details

**Table:**

Geometry. All esds (except the esd in the dihedral angle between two l.s. planes) are estimated using the full covariance matrix. The cell esds are taken into account individually in the estimation of esds in distances, angles and torsion angles; correlations between esds in cell parameters are only used when they are defined by crystal symmetry. An approximate (isotropic) treatment of cell esds is used for estimating esds involving l.s. planes.

### Fractional atomic coordinates and isotropic or equivalent isotropic displacement parameters (Å^2^)

**Table:**

	*x*	*y*	*z*	*U*_iso_*/*U*_eq_	
Br1	0.79020 (5)	0.53149 (2)	0.04905 (9)	0.02338 (12)	
S1	0.84814 (9)	0.70527 (2)	0.54322 (17)	0.01212 (17)	
S2	0.64506 (12)	0.70791 (2)	0.95068 (14)	0.01322 (19)	
C1	0.7514 (4)	0.73205 (10)	0.7447 (6)	0.0120 (7)	
C2	0.6459 (5)	0.66817 (10)	0.8081 (6)	0.0122 (8)	
H2	0.583254	0.648059	0.861560	0.015*	
C3	0.7350 (5)	0.66579 (9)	0.6225 (6)	0.0117 (8)	
C4	0.7515 (4)	0.63362 (9)	0.4874 (6)	0.0125 (8)	
C5	0.6886 (4)	0.60030 (9)	0.5600 (9)	0.0159 (7)	
H5	0.637106	0.598599	0.699223	0.019*	
C6	0.7004 (5)	0.56997 (10)	0.4326 (7)	0.0182 (8)	
H6	0.658485	0.547656	0.484660	0.022*	
C7	0.7737 (5)	0.57250 (10)	0.2289 (7)	0.0148 (8)	
C8	0.8384 (4)	0.60494 (10)	0.1519 (6)	0.0133 (7)	
H8	0.890749	0.606365	0.012995	0.016*	
C9	0.8253 (4)	0.63529 (10)	0.2817 (6)	0.0129 (8)	
H9	0.867405	0.657541	0.228922	0.015*	

### Atomic displacement parameters (Å^2^)

**Table:**

	*U*^11^	*U*^22^	*U*^33^	*U*^12^	*U*^13^	*U*^23^
Br1	0.0322 (2)	0.01276 (17)	0.02519 (19)	−0.00010 (14)	0.0039 (2)	−0.0046 (2)
S1	0.0126 (4)	0.0117 (4)	0.0120 (4)	−0.0005 (3)	0.0032 (5)	0.0006 (5)
S2	0.0148 (4)	0.0149 (4)	0.0100 (4)	0.0001 (4)	0.0030 (4)	0.0018 (4)
C1	0.0071 (15)	0.0181 (17)	0.0109 (16)	0.0014 (14)	0.0010 (12)	0.0008 (15)
C2	0.0111 (17)	0.0111 (19)	0.0145 (18)	−0.0007 (13)	−0.0011 (14)	0.0022 (14)
C3	0.0089 (17)	0.0126 (19)	0.0134 (18)	0.0024 (13)	−0.0025 (12)	0.0042 (13)
C4	0.0074 (15)	0.0130 (18)	0.017 (2)	0.0019 (12)	−0.0022 (12)	0.0010 (13)
C5	0.0136 (15)	0.0191 (17)	0.0149 (16)	0.0002 (12)	0.0022 (19)	0.002 (2)
C6	0.022 (2)	0.0124 (19)	0.020 (2)	0.0007 (15)	0.0009 (16)	0.0067 (17)
C7	0.0141 (18)	0.0119 (19)	0.0186 (19)	0.0014 (14)	−0.0036 (16)	−0.0017 (16)
C8	0.0121 (18)	0.0160 (19)	0.0117 (17)	−0.0007 (14)	0.0003 (15)	0.0017 (15)
C9	0.0115 (17)	0.0127 (19)	0.0144 (18)	0.0007 (14)	−0.0014 (14)	0.0029 (15)

### Geometric parameters (Å, º)

**Table:**

Br1—C7	1.901 (4)	C4—C9	1.394 (5)
S1—C1	1.762 (4)	C5—H5	0.9500
S1—C3	1.778 (4)	C5—C6	1.385 (6)
S2—C1	1.760 (4)	C6—H6	0.9500
S2—C2	1.729 (4)	C6—C7	1.384 (6)
C1—C1^i^	1.343 (7)	C7—C8	1.394 (5)
C2—H2	0.9500	C8—H8	0.9500
C2—C3	1.338 (5)	C8—C9	1.396 (6)
C3—C4	1.472 (5)	C9—H9	0.9500
C4—C5	1.409 (5)		
			
C1—S1—C3	94.28 (17)	C6—C5—C4	121.4 (4)
C2—S2—C1	93.94 (18)	C6—C5—H5	119.3
S2—C1—S1	114.5 (2)	C5—C6—H6	120.3
C1^i^—C1—S1	124.66 (13)	C7—C6—C5	119.4 (4)
C1^i^—C1—S2	120.87 (12)	C7—C6—H6	120.3
S2—C2—H2	120.1	C6—C7—Br1	120.5 (3)
C3—C2—S2	119.9 (3)	C6—C7—C8	120.9 (4)
C3—C2—H2	120.1	C8—C7—Br1	118.6 (3)
C2—C3—S1	115.3 (3)	C7—C8—H8	120.5
C2—C3—C4	125.9 (3)	C7—C8—C9	119.1 (3)
C4—C3—S1	118.7 (2)	C9—C8—H8	120.5
C5—C4—C3	120.9 (3)	C4—C9—C8	121.3 (3)
C9—C4—C3	121.2 (3)	C4—C9—H9	119.3
C9—C4—C5	117.9 (4)	C8—C9—H9	119.3
C4—C5—H5	119.3		

Symmetry code: (i) *x*, −*y*+3/2, *z*.
